# High-throughput sequencing reveals differential expression of miRNAs in prehierarchal follicles of laying and brooding geese

**DOI:** 10.1152/physiolgenomics.00011.2016

**Published:** 2016-05-06

**Authors:** Jing Yu, Ke He, Ting Ren, Yaping Lou, Ayong Zhao

**Affiliations:** College of Animal Science and Technology, Zhejiang A&F University, Lin'an, Zhejiang Province, China

**Keywords:** high-throughput sequencing of miRNA, folliculogenesis, goose follicle, brooding and laying

## Abstract

Broodiness is the primary factor influencing egg production in geese, in which several genes and miRNAs participate. Detailed spatiotemporal profiles of miRNAs encompassing follicle development levels, however, are lacking. In this study, we collected preovulatory follicles (classified as small white follicles, large white follicles, and small yellow follicles) from brooding and laying geese and aimed to analyze microRNA (miRNA or miR) during folliculogenesis. High-throughput sequencing and bioinformatics analysis were used to identify the miRNAs involved in follicle development. The let7 family, miR-10 family, and miR-143 family were abundant in these libraries, and they have been suggested to play a housekeeping role during folliculogenesis. Joint comparisons revealed 23 upregulated and 21 downregulated miRNAs (in at least two comparisons of follicles during brooding and laying, *P* < 0.1) in the laying stage. Unlike reproduction pathways reported for ovaries, GO and KEGG analysis suggested pathways for cell apoptosis and proliferation, such as the regulation of actin cytoskeleton, endocytosis, axon guidance, pathways in cancer, tight junctions, focal adhesion, the MAPK signaling pathway, cytokine-cytokine receptor interactions, and the Wnt signaling pathway in folliculogenesis. This study revealed the miRNAs that were directly involved in follicular atresia, and our results added to the understanding of the functional involvement of miRNAs during specific stages of follicle development.

avians have thousands of follicles in the ovary, and a strictly controlled follicular hierarchy is maintained, which is established through development, selection, and atresia in the follicles (24). As with other vertebrates, only a small percentage of follicles can be selected, reach maturity, and undergo ovulation. However, after a single follicle is selected for final maturation and ovulation, there is little atresia associated with the unselected follicles within the avian prehierarchal cohort, which will be included for selection of a subsequent follicle ∼24 h later (15). This well-organized follicular hierarchy controls the amount of egg production in birds until the ceasing period arrives, when the rest of the prehierarchal follicles atrophy. It has been suggested that the follicular atresia is triggered by granulosa cell (GC) apoptosis, and they cannot return to normal development once atresia occurs (13).

In avians, the homeostasis of selection and atresia in follicles is primarily orchestrated by synergistic interactions between extraovarian signals of hypothalamic-pituitary-gonadal origin and locally secreted growth factors. Certain hormones, such as follicle-stimulating hormone, anti-Müllerian hormone (14), and gonadotrophin-releasing hormone, all participate in the process. For the gene regulation network, microRNAs (miRNAs or miRs) have been shown to have a function in follicle development, which is regulated at the posttranscriptional level. Several studies on humans (29), pigs (18), goats (11), sheep (22), chickens (16), ducks (35), bovines (27, 30), and geese (3, 33) have already reported specific miRNAs with different expressions related to ovarian folliculogenesis. In 2015 Cao et al. (1) showed that let-7g increased the apoptotic rate of cultured GC. Conversely, miR-92 could regulate GC apoptosis in pig ovaries by targeting the Smad7 gene directly, and it can be considered as an inhibitor of GC apoptosis (20).

Most common avian species have production cycles including laying and brooding stages. An unresolved question in birds pertains to the identity of the most proximal factor that mediates seasonal follicular atresia. In this study, we chose geese as the bird to be studied. They have low fecundity and seasonal breeding. This means that many follicles that cannot pass the selection process will diminish via apoptosis originating within the GC layer during the brooding stage. Once the seasonal breeding mechanism is well resolved, poultry production of geese might increase significantly. Until now, attention has been devoted to the miRNAs in ovaries of laying and broody geese (21, 33), which has revealed the endocrine state of the ovary. However, little attention has been given to what happens in prehierarchal follicles, and there have not been detailed spatiotemporal profiles described that encompass several follicle development levels of both brooding and laying stages. It is believed that atresia happens in follicles of all sizes, but the probability of atresia varies with follicles at different levels. The prehierarchal follicles are considered to be susceptible to atresia, where a follicle that has been selected into the preovulatory follicle hierarchy has low opportunity for atresia (14), and the key factor for follicles to be selected into a hierarchical sequence is being 6–10 mm in diameter (6). Therefore, in this study, we obtained prehierarchal follicles at different levels during the brooding and laying stages [small white follicles (SWF) 2–4 mm, large white follicles (LWF) 4–6 mm, and small yellow follicles (SYF, 6–8 mm); these follicles can be separated according to diameter and color, following the criteria described in Ref. 9], constructed small RNA libraries, and identified miRNAs differentially expressed in the follicles of laying and brooding geese using high-throughput technologies and deep sequencing analysis. The results may establish the spatiotemporal pattern of significant miRNAs in follicle development and reveal some useful information about follicle apoptosis in geese reproduction.

## MATERIALS AND METHODS

### 

#### Animals and samples.

All of the following procedures were approved by the Institutional Animal Care and Use Committee of Zhejiang University (Hangzhou, China). Zhedong geese were selected from the Xiangshan goose national conservation farm (Xiangshan, China). This bird has strong broodiness and shows broodiness once the laying period is over, which happens four times per year. We selected optional individuals from a half-sib family of nearly 100 and determined the brooding and laying stages of geese by assessing: *1*) the egg laying record (geese start to show incubating behavior after having laid approximately 8–10 eggs with an interval of 3–4 days), *2*) the experience of farmers (who can judge whether there were postovulatory follicles in the ovary by touching the belly), *3*) the morphology of follicles (the surface subsidence suggesting brooding), and *4*) the concentration of hormones of follicles and ovaries [including prolactin, progesterone (P4), and estradiol, for details see Yu et al. (35a)]. The sampling strategy is illustrated in Supplemental Fig. S1.^1^ Twenty geese at 380 days of age were killed by exsanguination, and ovarian samples were removed. SWF, LWF, and SYF were obtained quickly, frozen in liquid nitrogen, and stored at −80°C until use.

#### RNA extraction, library construction, and sequencing.

Each prehierarchal follicle level (SWF, LWF and SYF) at a brooding or laying stage (B or L) was represented by three samples, and each sample was treated independently. We used BSWF as the abbreviation for SWF at the brooding stage, and thus had BLWF, BSYF, LSWF, LLWF and LSYF categories. Total RNA was extracted using Trizol reagent (Invitrogen) and ∼1 μg of total RNA was used to prepare a small RNA library using TruSeq Small RNA Sample Prep Kits (Illumina, San Diego, CA). Then we performed single-end sequencing (36 bp) on an Illumina Hiseq2500 at LC-BIO (Hangzhou, China).

#### Sequence data analysis and identification of miRNAs.

Clean data sequences were isolated from the raw reads with ACGT101-miR (LC Sciences, Houston, TX). Subsequently, specific species precursors in the miRBase 20.0 (because there was no miRNA database for our species in the miRBase, we took gga as the specific species) and the genomes of goose (http:/www.ncbi.nlm.nih.gov/nuccore/AOGC00000000) were used to confirm known miRNAs and novel 3p- and 5p-derived miRNAs. Length variation at both 3′- and 5′-ends and one mismatch inside the sequence were allowed in the alignment. The hairpin RNA structures containing sequences of novel miRNAs were predicted from the flank 80 nt sequences using RNAfold software (http://rna.tbi.univie.ac.at/cgi-bin/RNAfold.cgi). The criteria for secondary structure prediction are as described in Ref. 23, except that the defining numbers for *criteria 6, 7*, and *9* are 8, 4, and 7.

#### Analysis and predicted target genes of differentially expressed miRNAs.

We analyzed differential expression of miRNAs by selectively using Fisher's exact test, the χ^2^ 2×2 test, the χ^2^ *n*×*n* test, Student's *t*-test, and an ANOVA based on the experimental design and normalized deep-sequencing counts. We set the significance threshold at 0.01 or 0.05 in each test.

#### Analysis by Gene Ontology and the Kyoto Encyclopedia of Genes and Genomes pathway.

To understand the biological function of significantly expressed miRNA in the follicles of geese, we predicted the putative target genes of miRNA by TargetScan 50 and miRanda 3.3a, which excluded genes with a contest score percentile <50 and a maximum energy >−10, respectively. We chose targeted genes of intersection for analysis. Gene Ontology (GO) and the Kyoto Encyclopedia of Genes and Genomes (KEGG) pathway were used to analyze target genes and pathways.

#### Validation of biological variability of a stage.

To minimize the effect of biological variability, the follicles for sequencing were from a half-sib family. After sequencing, we calculated the correlation of the clean data from three libraries at the same level and stage such as BSWF1, BSWF2, and BSWF4. Furthermore, to validate the reliability of the Illumina analysis, 10 differentially expressed identified miRNAs were selected randomly (Supplemental Table S1) and detected by quantitative real-time PCR (qRT-PCR). Follicle samples were taken from the same individuals of the constructed libraries to verify the sequencing results, and tissues (including spleen, liver, intestine, heart, lung and breast muscle) were obtained from three individual laying geese to determine whether follicle-specific miRNAs existed. Total RNA was extracted as described for the library preparation, and cDNA synthesis and qRT-PCR analyses were performed with Mir-X miRNA First-Strand Synthesis and SYBR qRT-PCR (TaKaRa, Dalian, China) on a Real-Time PCR Detection System, respectively. Three repetitions were made for each sample. U6 snRNA was taken as the control, and the quantification of each miRNA relative to the U6 gene was calculated by the equation: *n* = 2^−ΔΔCt^.

## RESULTS

### 

#### Validation of biological variability between samples of a stage.

Because biological variability was inevitable, relative coefficients were calculated with data from three libraries of follicles in the same level at the same stage. Most R values ranged from 0.817 to 0.988 (Supplemental Fig. S2), which indicates that the effect of biological variability was not significant in this study and that the data used were reliable. However, the consistency of the LSYF data was not high, with R values of 0.483 and 0.524 in LSYF12 vs. LSYF13 and LSYF11 vs. LSYF13. The R value of LSYF11 vs. LSYF12 was 0.987, so we discarded the sequencing results of LSYF13, as well as qPCR, in further analysis.

#### Overview of the sequencing results, identification of known and conserved miRNAs, and prediction of novel miRNAs expressed in the follicles of geese.

A total of 12,024,983; 11,320,973; 11,411,741; 12,524,050; 9,637,835; and 12,650,520 raw reads were obtained from BSWF, BLWF, BSYF, LSWF, LLWF, and LSYF libraries, respectively (Supplemental Table S2). After quality control and adapter removal, several databases such as Repeatbase (18.02) and Rfam (10.1) were used to filter non-miRNA sequences (Supplemental Table S2, Supplemental Fig. S3). Finally, we obtained clean data on miRNAs for further analysis. MiRNA occupied the highest proportion in the RNA species (both based on read counts and unique numbers), and the numbers were 6,902,098 (57.40%), 6,306,091 (55.70%), 5,297,086 (46.42%), 9,834,225 (78.52%), 6,911,841 (71.72%), and 7,182,785 (56.78%) for the clean reads available from BSWF, BLWF, BSYF, LSWF, LLWF, and LSYF libraries, respectively. This indicates that those libraries were highly enriched with mature miRNAs. Length distribution analysis showed that most abundant reads ranged from 20 to 24 nt with a percentages of 78.20, 74.07, 70.87, 89.02, 86.13, and 84.81% for BSWF, BLWF, BSYF, LSWF, LLWF, and LSYF libraries, respectively, and the most abundant size of miRNAs was 22 nt, ranging from 19.15 to 40.79% in the six libraries (Supplemental Fig. S4). This finding conforms to the typical characteristics of digestion with Dicer enzymes. All of the results above indicate the data were suitable for analysis of miRNA expression profiling during follicle development.

The sequences were divided into five groups according to whether they mapped to specific or selected miRNAs/pre-miRNAs in the miRbase and the pre-miRNAs mapped to genome and expressed sequence tag (Table 1). A complete sequence, name, and relative abundance list for the 1,183 miRNA sequences detected in gp1-3 is provided in Supplemental Table S3. For novel miRNAs, most of the expression levels were low (the total number of reads is <10). Only the 178 novel miRNAs with middle and high expression are listed in Supplemental Table S4. The novel and candidate miRNAs were typically in low abundance compared with most of the known miRNAs, which may explain how they might have eluded previous detection efforts. Remarkably, one of the novel miRNAs (novel miR-144, miR sequence CCTGCCTGAGCGTCGCTT, detail in TS4) was counted up to 14,379 times in the BSYF library. It is hypothesized that this highly expressed novel miRNA may be specific to geese and may play a potential role in follicle development.

**Table 1.** Number of miRNAs in results of goose follicles

Group	miRNAs, *n*	gga-miRNAs/pre-miRNAs in miRbase	Other miRNAs/pre-miRNAs in miRbase*	Pre-miRNA Map to the Goose Genome
gp1a	412	√		√
gp1b	73		√	√
gp2	167	√		×
gp3	531	√		×
gp4	6010	×	×	

miRNA, microRNA.

* tgu, hsa, mmu, bta, eca, ppy, ptr, mml, cfa, efu, rno, mdo, oan, ssc, ssa, ggo, cin, dre, aca, ipu, chi, pma, cgr, oha, xtr, tch, loa, bfl, ccr, tni, fru, bbe, oar, ppa, mne, odi, sha, age, lla, sla, hhi, csa, xla, pol, aja, lca, ocu, pbi, ssy, xbo, and mues as selected species.

#### Known and conserved miRNAs highly expressed in the follicles of geese.

Our first objective was to explore the miRNAs with important roles in follicle development from the expression profiling of identified miRNA populations. A closer look at the known miRNAs from the six libraries in this study showed that, in the groups of catalogued known and conserved miRNAs, there were 113 miRNAs that had a high expression level. We list the top 20 abundant miRNAs in Table 2. There are 13 miRNAs listed in the top 20 of the six libraries (but with a different order). It is suggested that the miRNAs play a housekeeping role in maintaining normal physiological functions at various levels and stages of these miRNAs. Six miRNAs were only detected in one library's top 20 list (aca-miR-191-5p and gga-miR-30e-5p in LSWF, gga-miR-205a in BSWF, gga-miR-30d in LSYF, and mmu-miR-145a-5p in LLWF), which indicates special roles for these miRNAs in a specified period.

**Table 2.** Summary of top 20 miRNAs in six libraries and related roles in folliculogenesis

miR Family	miR Name	Libraries	Reported Role/Changes during Development	Target Gene/Signal Pathway	Ref. List No.
let-7	gga-let-7b	6	participate in angiogenesis	*TIMP1*	25
			inhibited testosterone release		29
miR-10	gga-miR-100-5p	6			
miR-10	gga-miR-10a-5p	6			
miR-10	gga-miR-125b-5p	6	decrease during luteinization	(*LIF, CDKN1A*)*	22
miR-148	gga-miR-148a-3p	6	related to ovarian cancer		8
miR-199	gga-miR-199-5p	6	related to ovarian cancer		10
miR-21	gga-miR-21-5p	6	related to GC apoptosis, and highly induced by LH		2
			related to follicular-luteal transition		11
miR-22	gga-miR-22-3p	6	related to the diminished ovarian reserve		5
miR-26	gga-miR-26a-5p	6	inhibited testosterone release		29
miR-30	gga-miR-30c-5p	6	functions as a tumor suppressor	oncogenic Wnt/β-catenin/BCL9 pathway	38
miR-25	gga-miR-92-3p	6	as biomarker of benign and ovarian cancer patients		28
miR-10	gga-miR-99a-5p	6			
miR-143	mmu-miR-143-3p	6	inhibited primordial follicle formation	*CDKs4*, *CDKs6*, cyclins B1, D2, and E2	36
			promoted progesterone release		29
let-7	gga-let-7a-5p	5 (BSYF)†	inhibited testosterone release		29
			downregulated during follicle atresia		31
let-7	gga-let-7i	5 (LSWF)	related to ovarian cancer		28
miR-126	gga-miR-126-3p	6	as biomarker of benign and ovarian cancer patients		28
			regulation in CL during the estrous cycle	*TLN2*	4
miR-199	gga-miR-199-3p	5 (LSYF)	related to follicular-luteal transition		22
miR-181	gga-miR-181a-5p	5 (LSWF)	inhibits ovarian GC proliferation	*acvr2a*	37
let-7	gga-let-7f-5p	3 (LSWF, LLWF, BSWF)			
let-7	gga-let-7 g-5p	3(LSWF, LLWF, LSWF)	GCs apoptosis	TGFBR1/MAP3K1/TGF-β signaling pathway	1
miR-214	gga-miR-214	3 (BSYF, BLWF, BSWF)			
miR-2954	gga-miR-2954	3 (BSYF, LSYF, BLWF)			29
miR-191	aca-miR-191-5p	1 (LSWF)	promoted progesterone release in GC		
miR-205	gga-miR-205a	1 (BSYF)			
miR-30	gga-miR-30d	1 (LSYF)	regulated by FSH		34
miR-30	gga-miR-30e-5p	1 (LSWF)			
miR-145	mmu-miR-145a-5p	1 (LLWF)	decrease during luteinization	(*CDKN1A*)	22
			promoted progesterone release in GC		29

* The targeted genes were predicted by bioinformatics analysis rather than being verified in vivo or vitro.

† For miRNAs (also abbreviated as miR) in 5 libraries, we list the absent library in parentheses, and for miRNAs in 3 libraries or only 1 library, the existing libraries are listed.

B, brooding stage; L, laying stage; SWF, small white follicle; LWF, large white follicle; SYF, small yellow follicle.

#### Differential expression of miRNAs between laying and brooding geese.

**Fig. 1.**
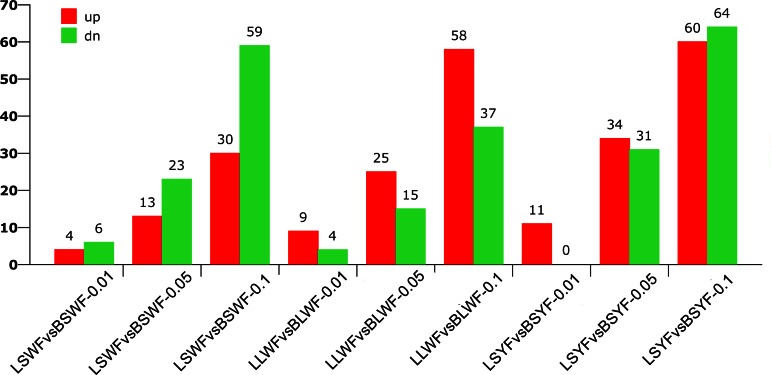
Histogram of differentially expressed microRNAs (miRNAs or miRs) between brooding (B) and laying (L) follicles. SWF, small white follicle; LWF, large white follicle; SYF, small yellow follicle.

Apart from the abundant miRNAs acting with a housekeeping role, miRNAs with significant expression regulation during different stages were also noticeable in miRNA sequencing. We compared the follicles at the same level but of different status to search for miRNAs related to atresia (BSWF vs. LSWF, BLWF vs. LLWF, and BSYF vs. LSYF). The significant expression profiles of the libraries are shown in Supplemental Table S5, and the numbers are illustrated in Fig. 1. We confirmed 45 miRNAs including eight novel miRNAs to have differential expression in at least two comparisons (Table 3). When analyzing the expression levels, we took results from follicles at the laying stage as the numerator and results from follicles at the brooding stage as the denominator. There were 23 upregulated miRNAs, 21 downregulated miRNAs, and one uncertain miRNA in this list. Expression in bta-miR-652 was inconsistent, which might be caused by the low expression of this miRNA, so we did not adopt it. Furthermore, five miRNAs (gga-miR-130a-5p, gga-miR-1416-3p, mmu-miR-143-5p, oha-miR-1d-3p, and novel-miR-93) had the same up or down expression pattern in three levels of follicles when brooding was compared with laying stages, which indicates they might participate in follicle atresia at each level.

**Table 3.** List of identified miRNAs having different expression in at least two comparisons

			LSWFvs. BSWF		LLWFvs. BLWF		LSYFvs. BSYF	
miR Name	Up/Down	Expression Level	Fold	*P* Value	Fold	*P* Value	Fold	*P* Value
aca-miR-10c-3p	up	M			1.93	**	2.32	**
aca-miR-31-5p	up	M	2.53	*	3.14	***		
bta-miR-342	down	M	0.04	*			-inf	*
bta-miR-652	uncertain	L			inf	***	0.10	**
chi-miR-16b-5p	down	M	0.12	*			0.25	*
csa-let-7d	down	H			0.51	**	0.55	**
gga-let-7 g-5p	up	H	1.48	**	1.85	**		
gga-miR-100-3p	down	M			0.46	*	0.10	*
gga-miR-130a-5p	up	M	2.21	**	7.16	*	3.38	***
gga-miR-1329-3p	up	M			2.11	*	2.76	**
gga-miR-140-5p	up	H	1.85	*	2.40	***	1.46	**
gga-miR-1416-3p	up	M	2.61	**	2.19	**	5.50	***
gga-miR-1451-5p	down	H	0.70	*	0.72	*		
gga-miR-146a-5p	down	H			0.50	**	0.50	**
gga-miR-153-3p	down	M			0.58	*	0.40	*
gga-miR-15b-5p	down	M	0.47	*			0.40	*
gga-miR-15c-5p	down	M	0.30	**			0.22	**
gga-miR-1677-3p	down	M	0.50	***			0.17	*
gga-miR-183	down	M			0.45	*	0.38	**
gga-miR-187-5p	up	M	2.46	*	2.71	*		
gga-miR-1a-3p	up	H			2.95	*	1.94	**
gga-miR-200b-3p	up	H			2.13	**	1.63	**
gga-miR-200b-5p	up	M			2.16	***	2.06	***
gga-miR-221-3p	up	H			1.94	**	2.23	***
gga-miR-26a-3p	up	M			1.92	**	1.47	**
gga-miR-301a-5p	up	M	4.02	**			5.97	*
gga-mir-3535-p3	down	M	0.22	*			0.32	**
gga-miR-455-3p	down	M	0.52	***	0.50	***		
gga-miR-460b-5p	up	M			2.45	**	1.91	*
hsa-miR-16-5p	down	M	0.17	**	0.19	**		
hsa-miR-338-5p	up	M	1.65	*			1.75	**
mmu-miR-145a-3p	up	H			1.61	*	1.53	*
mmu-miR-143-5p	up	H	1.69	***	1.95	*	1.68	*
oha-miR-1d-3p	up	M	8.31	*	12.31	**	8.97	**
tgu-miR-125-5p	up	H			1.34	*	1.65	*
tgu-miR-139-5p	down	M	0.55	*			2.75	**
tgu-miR-454-5p	up	M	2.37	**			5.44	*
novel-miR-116	down	M	0.04	**			0.02	*
novel-miR-126	down	M	0.02	***	0.03	**		
novel-miR-13	up	M			1.57	**	2.53	*
novel-miR-146	down	M	0.01	*	0.02	**		
novel-miR-158	up	M			inf	*	inf	**
novel-miR-47	down	M			0.49	**	0.14	*
novel-miR-62	down	M	0.12	*			0.29	*
novel-miR-93	down	M	0.01	*	-inf	*	0.10	*

M, middle; L, low; H, high; inf, norm average of reads in brooding follicles is too low to be detected; -inf, norm average of reads in laying follicles is too low to be detected.

The *P* values were calculated by *t*-test.

* *P* < 0.1,

** *P* < 0.05 and

*** *P* < 0.001.

We also paid attention to the analysis of different expression profiles of BSWF vs. BLWF vs. BSYF and LSWF vs. LLWF vs. LSYF (Supplemental Table S5), which can reveal miRNAs related to follicle development. We found 10 miRNAs to have a significant different expression pattern in both comparisons (Table 4). Of this list, five miRNAs had lowest expression in LWF (dre-miR-92a-3p, gga-miR-3535-p3, gga-miR-3538-p3, gga-miR-3538-p3 and novel-miR-117), one miRNA (efu-miR-423) had increasing expression during development of follicles, one miRNA (gga-miR-32-5p) had highest expression in LWF, tgu-miR-2970-3p had highest expression in SYF, and two miRNAs had a different expression pattern. This revealed some potential miRNAs that might play a role in the folliculogenesis of different grades (further work is needed for information on the transcriptome, which is in progress).

**Table 4.** List of identified miRNAs with different expression in comparisons of LSWF vs. LLWF vs. LSYF and BSWF vs. BLWF vs. BSYF

		LSWF vs. LLWF vs. LSYF			BSWF vs. BLWF vs. BSYF		
miR Name	Expression Level	LSWF/LLWF	LLWF/LSYF	*P* Value	BSWF/BLWF	BLWF/BSYF	*P* Value
dre-miR-92a-3p	H	1.26	0.34	**	1.22	0.36	*
efu-miR-423	M	0.39	0.23	*	0.51	0.11	***
gga-miR-32-5p	M	0.94	2.89	**	0.65	2.12	*
gga-miR-3535-p3	M	1.57	0.28	*	1.09	0.40	**
gga-miR-3538-p3	M	3.08	0.06	***	1.88	0.19	**
mmu-miR-486a-5p	M	1.13	0.26	***	1.99	0.20	**
ssa-miR-221-5p	M	1.68	0.29	**	0.95	5.14	*
tgu-miR-139-5p	M	1.42	0.31	**	0.60	1.58	**
tgu-miR-2970-3p	M	0.99	0.42	*	1.28	0.41	*
novel-miR-117	M	1.13	0.33	**	1.68	0.22	*

The *P* values were calculated by ANOVA.

#### Validation of identified miRNAs.

**Fig. 2.**
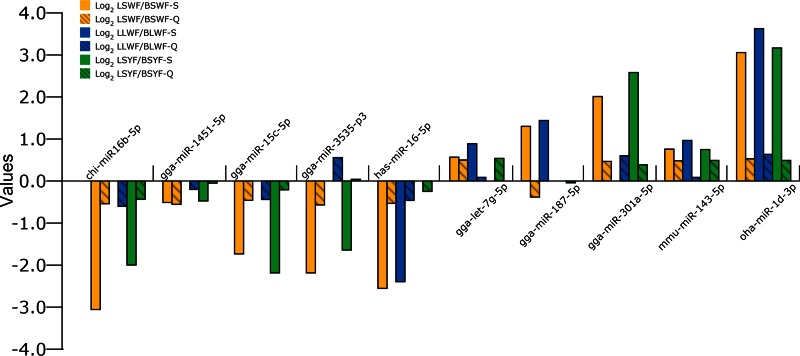
The sequencing (S) and quantitative PCR (Q) results of 10 miRNAs in follicles. SWF, LWF, and SYF are illustrated in yellow, blue, and green, respectively; the results of Q are represented with a columnar line. We took the fold values as 1 when significant expression was not found in comparisons with the results of sequencing.

**Fig. 3.**
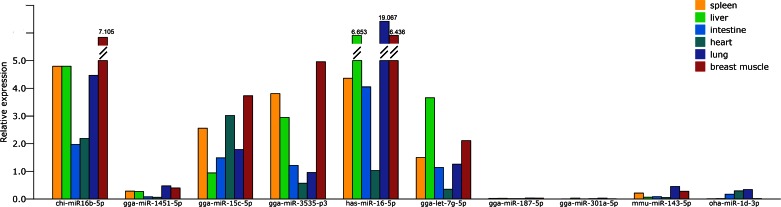
The qPCR results of 10 miRNAs in tissues.

qRT-PCR analysis of 10 goose miRNAs was performed in follicles at brooding and laying stages (Fig. 2). Eight miRNAs were in agreement with the expression pattern found in the high-throughput sequencing data, indicating the sequenced data and analysis methods were reliable. For the miRNAs with different expression (gga-miR-3535-p3 and gga-miR-187-5p), low fold values (LSYF/BSYF = 1.025 in gga-miR-3535-p3 and LSWF/BSWF = 0.766 in gga-miR-187-5p) and low expression (no more than 1,000 reads) might explain this phenomenon. qRT-PCR was also performed in different tissues (Fig. 3). Five miRNAs had high expression in tissues, three miRNAs had low expression (gga-miR-1451-5p, mmu-miR-143-5p, and oha-miR-1d-3p), and two miRNAs were difficult to detect (gga-miR-187-5p and gga-miR-301a-5p). This indicates some miRNAs might specifically express in tissues; however, none were follicle specific.

#### Target gene prediction, GO enrichment, and KEGG pathway analysis.

**Fig. 4.**
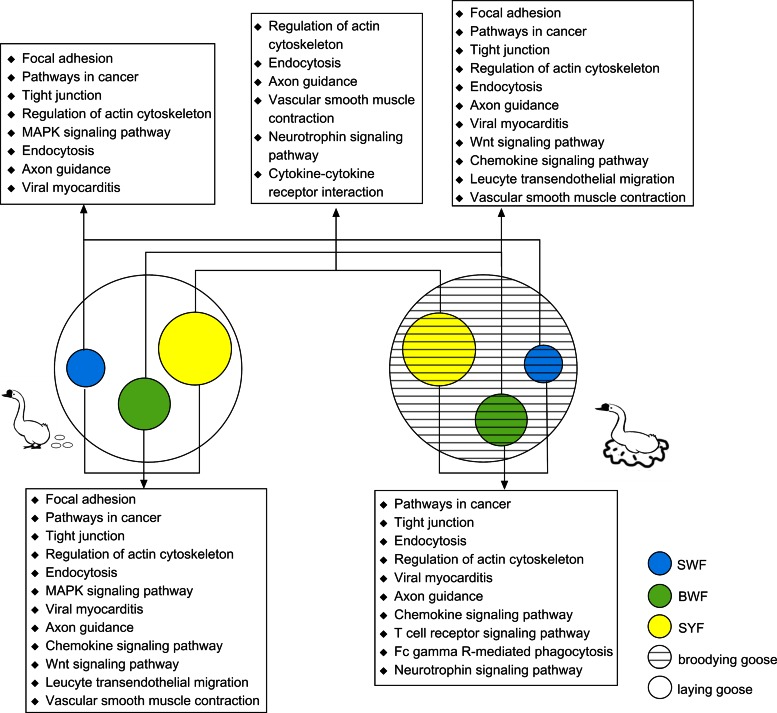
KEGG pathway analysis of significant expression miRNAs in follicular development.

Target prediction shows that the selected miRNAs of BSWF vs. LSWF, BLWF vs. LLWF, and BSYF vs. LSYF (which are listed in Supplemental Table S5, the miRNAs with high and middle expression level, fold change >2 and *P* < 0.05 were selected) corresponded to 3,759; 3,361; and 3,879 target genes, respectively. These targeted genes were classified according to KEGG function annotations to identify the pathways that were actively regulated by miRNAs in goose follicles, and they are thought to be involved in 62, 50 and 39 pathways, respectively (Fig. 4 Supplemental Table S6). We also performed GO and KEGG analysis of the miRNAs BSWF vs. BLWF vs. BSYF and LSWF vs. LLWF vs. LSYF (which are listed in Supplemental Table S5, the miRNAs with high and middle expression level, and *P* < 0.05 were selected). The numbers of targeted genes are 5,116 and 2,918 for two lists of miRNAs. We compared the top 20% pathways in five lists, which were arranged by the number of targeted genes (Fig. 4). Three pathways are involved in regulation of the actin cytoskeleton, endocytosis, and axon guidance, which all contained a high number of targeted genes in the five lists; pathways in cancer and tight junctions are listed in the forefront of four lists, except for LLWF vs. BLWF; other pathways, such as focal adhesion, the MAPK signaling pathway, cytokine-cytokine receptor interactions, the Wnt signaling pathway, and neurotrophin signaling pathway all play important roles in follicle development.

## DISCUSSION

MiRNAs are considered key regulators of gene expression at the posttranscriptional level. In this study, we aimed to investigate the roles of miRNAs in follicle atresia of geese, which could reveal detailed spatiotemporal profiles of follicle development. Six small RNA libraries were constructed, and each library of the same status contained three samples, which were treated independently. We detected 1,179 known conserved miRNAs and 178 novel miRNAs in goose follicles. The top 20 abundant miRNAs were shown to have important roles in regulating folliculogenesis. Joint analysis of three levels of follicles between brooding and laying stages showed 23 upregulated miRNAs and 21 downregulated miRNAs (*P* < 0.1). GO and KEGG analysis revealed that regulation of the actin cytoskeleton, endocytosis, axon guidance, pathways in cancer, and tight junctions all play vital roles in follicle development.

### 

#### MiRNAs with a housekeeping role in follicle development.

We identified miRNAs with important roles during follicle development using detailed spatiotemporal profiles. We reasoned that we could do this by comparing the miRNAs profiles of six libraries including different levels of follicles during brooding and laying stages. The ovary is important in reproduction because it provides the internal environment for follicle development. In 2014 Chen et al.(3) and Xu et al. (33) both investigated the miRNAs of the ovary in brooding and laying geese, and the same studies were undertaken in chickens (16) and ducks (35). However, these authors mostly focus on the expression of miRNAs in the ovary rather than follicles. The only published study of miRNAs in follicular development was done on sheep (22). Although there are different processes of follicle development between avians and mammals (such as follicle-luteal transition), the basic role of GC function (including steroidogenesis, proliferation, survival, terminal differentiation, and cumulus expansion) are similar, so we compared the results of sheep with ours. More than half of the miRNAs (10/18: miR-125b-5p, let-7a-5p, let-7b, let-7f-5p, let-7i, miR-199a-3p, miR-30c, miR-99a, miR-126, and miR-143), which represents 70% of the total miRNAs expressed in sheep, are listed in the top 20 miRNAs table for goose follicles. The consistency of abundant miRNAs in follicles in sheep and goose suggests a housekeeping role for these miRNAs.

After literature mining, we found that the miRNAs of the top 20 list are involved in regulating aromatase expression during follicle development, which is related to follicle development and survival, GC apoptosis, and ovarian cancer (Table 2). For example, the let-7 family was discovered to have the highest number of reads in our results, and five miRNAs of this family had high expression across the different development stages of the goose follicle. It was demonstrated that let-7a, let-7b, and let-7c inhibit the release of P4 and testosterone (19, 29); let-7i was thought to be a diagnostic biomarker in the ovary (28), and let-7g could promote follicular apoptosis in GC by targeting TGF-βR1 in porcine (1). The other highly expressed miRNAs were considered to be related to ovarian cancer, GC apoptosis, diminished ovarian reserve, and the release of hormones (details in Table 2), such as miR-143, which was suggested as inhibiting primordial follicle formation by targeting CDK 4 and CDK 6 and cyclins B1 D2, and E2 in pregranulosa cells (36), and miR-181a-5p, which targets activin receptor IIA (acvr2a) and can result in proliferating cell nuclear antigen expression, leading to inhibition of cellular proliferation (37). What most interests us in Table 2 are some miRNAs related to ovarian cancer and as biomarkers of ovarian cancer. It is well known that the loss of normal regulation of cell proliferation and differentiation will lead to abnormal proliferation and differentiation, which may occur in cancer. The same function might be played in follicle development; in other words, these housekeeping miRNAs might maintain normal cell life in follicles.

#### MiRNAs with differential expression between laying and brooding geese.

Apart from the abundant miRNAs acting with a housekeeping role, miRNAs with significant expression regulation during different stages were also noticeable in miRNA sequencing. We analyzed expression comparisons at two levels: between brooding and laying follicles (LSWF vs. BSWF, LLWF vs. BLWF, and LSYF vs. BSYF) and between different levels of follicles of the same status (LSWF vs. LLWF vs. LSYF and BSWF vs. BLWF vs. BSYF), which suggested miRNAs that are related with follicle atresia and development, respectively. Because nearly 100 miRNAs could be found in each analysis of brooding and laying follicles, we assessed the miRNAs with significant expression in at least two. In summary, 23 miRNAs were upregulated and 21 miRNAs were downregulated in the laying stage (Table 3). Remarkably, two miRNAs (gga-miR-3535-p3 and tgu-miR-139-5p) were both detected in the comparisons of the two levels (Tables 3 and 4). Krishnan et al. (17) suggested a potential role for miR-139-5p in regulating the ability of cells to invade and migrate. A similar role might be carried out by this miRNA in folliculogenesis.

We compared the miRNAs that were differentially expressed in our study (listed in Table 3) with results reported in Sontakke et al. (30), which compared small healthy vs. large healthy and large atretic vs. large healthy follicles in bovines, and only miRNAs with fold ≥2 were taken. Unfortunately, the two studies have only five miRNAs in common (2/16 in small vs. large and 3/57 in atretic vs. healthy), which reveals that the dynamic changes of miRNAs in mammals and avians were significantly different. We also assessed the miRNAs with significant expression regulation in the ovary and follicles in an analysis together, which revealed some consistency in the environment and endocrine status of follicles (Supplemental Table S7). We found 11 miRNAs with the same regulation in the Xu et al. study (33) and our studies; these are suggested to inhibit progesterone release (miR-15a-3p), promote P4 release in GCs (miR-182-5p) (29), suppress the expression of ZEB1 in the pituitary gland (miR-200b) (26), and relate to polycystic ovarian syndrome (PCOS) in humans (miR-185-5p) (32). Seven miRNAs were confirmed to have the same expression pattern in the ovary, but a different one in follicles. These miRNAs with identical regulation in the ovary are considered to be involved in oocyte maturation (miR-100b) (19), hormonal regulation in ovulation (miR-132) (7), and ovarian cancer (miR-140) (10). The standard for the three studies was different, which might be the main reason for these results (3, 33). However, to a certain degree, these results suggest significant miRNAs in both the ovary and follicle.

#### Targeted pathway of follicle development in geese.

Pathways were identified by KEGG function annotations. This result suggests that follicle regulation is different from that of ovaries in laying and broody geese (3, 33). It was reported that pathways for reproduction regulation are differently regulated in ovaries (21). In our study, we found some difference; e.g., three pathways for the regulation of actin cytoskeleton, endocytosis, and axon guidance all contained a high number of targeted genes in five lists; pathways for cancer and tight junctions are at the forefront of four lists, except for LLWF vs. BLWF; other pathways such as focal adhesion, the MAPK signaling pathway, cytokine-cytokine receptor interactions, the Wnt signaling pathway, and neurotrophin signaling pathway all played important roles in follicle development. Some pathways with regulatory functions, such as steroid hormone biosynthesis and steroid biosynthesis, were not found in the results of follicular KEGG analysis. Therefore, it was suggested that ovaries participate in secretion and signal transduction of the regulatory factor, while the follicle is the place where apoptosis and cell proliferation occur. This aids our understanding of follicle development and reproduction and provides us with a way to improve reproductive performance more directly.

### Conclusions

In this study, the detailed spatiotemporal profiles of miRNA during avian follicle development has been reported for the first time, and miRNAs with abundant reads and significant expressions were analyzed. We found let-7, miR-10, miR-30, and miR-199 families played a housekeeping role in folliculogenesis. Joint comparisons revealed 23 upregulated and 21 downregulated miRNAs during the laying stage. GO and KEGG analysis suggested pathways for cell apoptosis and proliferation, such as the regulation of actin cytoskeleton, endocytosis, axon guidance, and pathways for cancer, tight junctions, focal adhesion, the MAPK signaling pathway, cytokine-cytokine receptor interactions, and Wnt signaling pathway in folliculogenesis. We also performed an analysis of the ovary and follicle, and the results reveal candidate miRNAs that were directly involved in follicular atresia. These results add to the understanding of the functional involvement of miRNAs during specific stages of follicle development.

## GRANTS

This project was funded by the National Natural Science Foundation of China (31372349).

## DISCLOSURES

No conflicts of interest, financial or otherwise, are declared by the author(s).

## AUTHOR CONTRIBUTIONS

J.Y., K.H., and A.Z. conception and design of research; J.Y., K.H., T.R., Y.L., and A.Z. performed experiments; J.Y., K.H., T.R., and Y.L. analyzed data; J.Y. and K.H. prepared figures; J.Y., K.H., and A.Z. drafted manuscript; J.Y., K.H., and A.Z. edited and revised manuscript; J.Y., K.H., T.R., Y.L., and A.Z. approved final version of manuscript; K.H. interpreted results of experiments.

## Supplementary Material

Figure S1

Figure S2

Figure S3

Figure S4

Table S1

Table S2

Table S3

Table S4

Table S5

Table S6

Table S7

## Footnotes

1 The online version of this article contains supplemental material.
