# Supplementary figures and images for: High-throughput sequencing reveals differential expression of miRNAs in prehierarchal follicles of laying and brooding geese
Source: Physiol Genomics. 2016 May 6;48(7):455–63. doi: 10.1152/physiolgenomics.00011.2016 (PMC4967221; doi:10.1152/physiolgenomics.00011.2016)

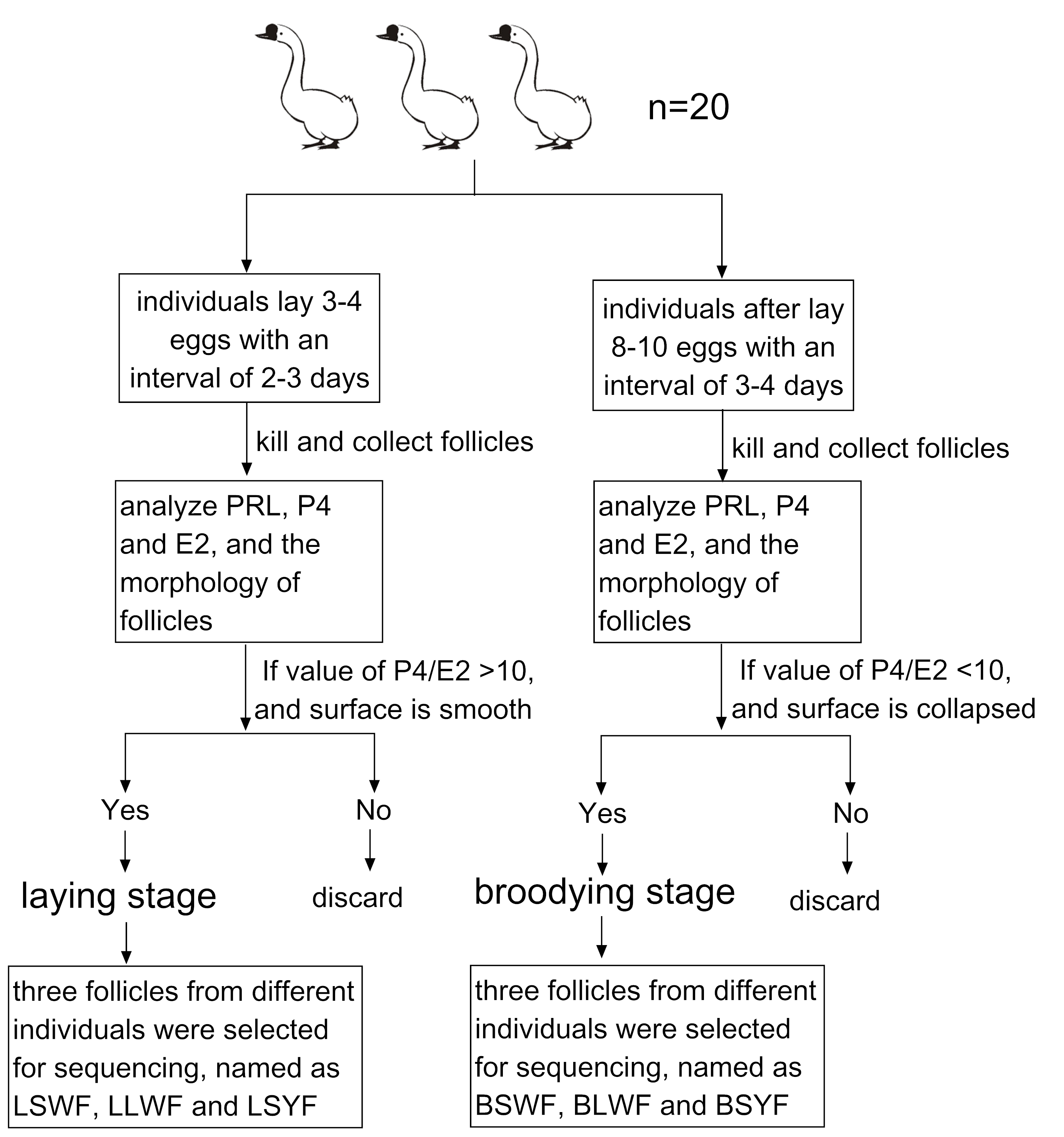

Supplement: Figure S1 [file Figure_S1.tif]

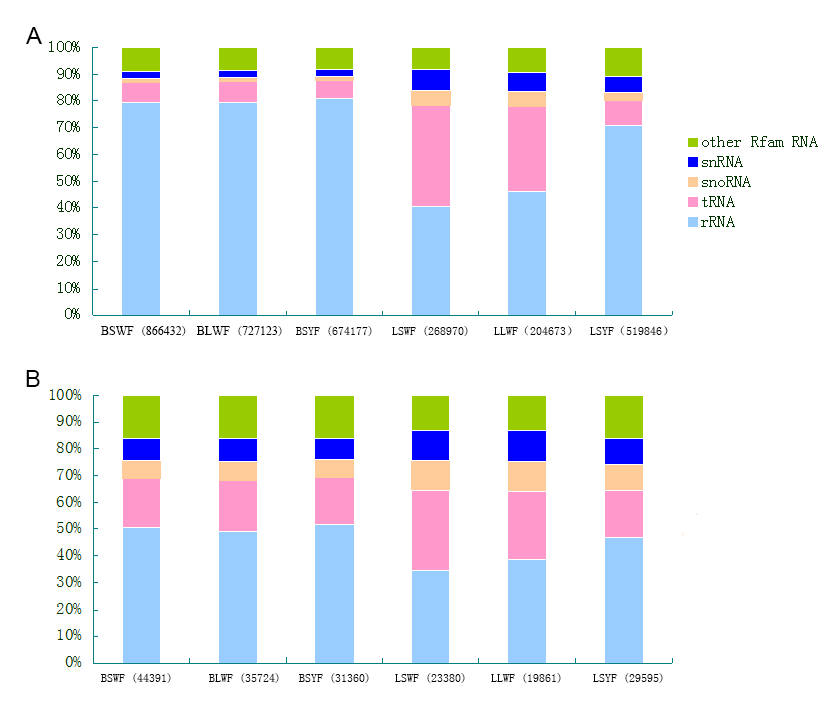

Supplement: Figure S3 [file Figure_S3.tif]

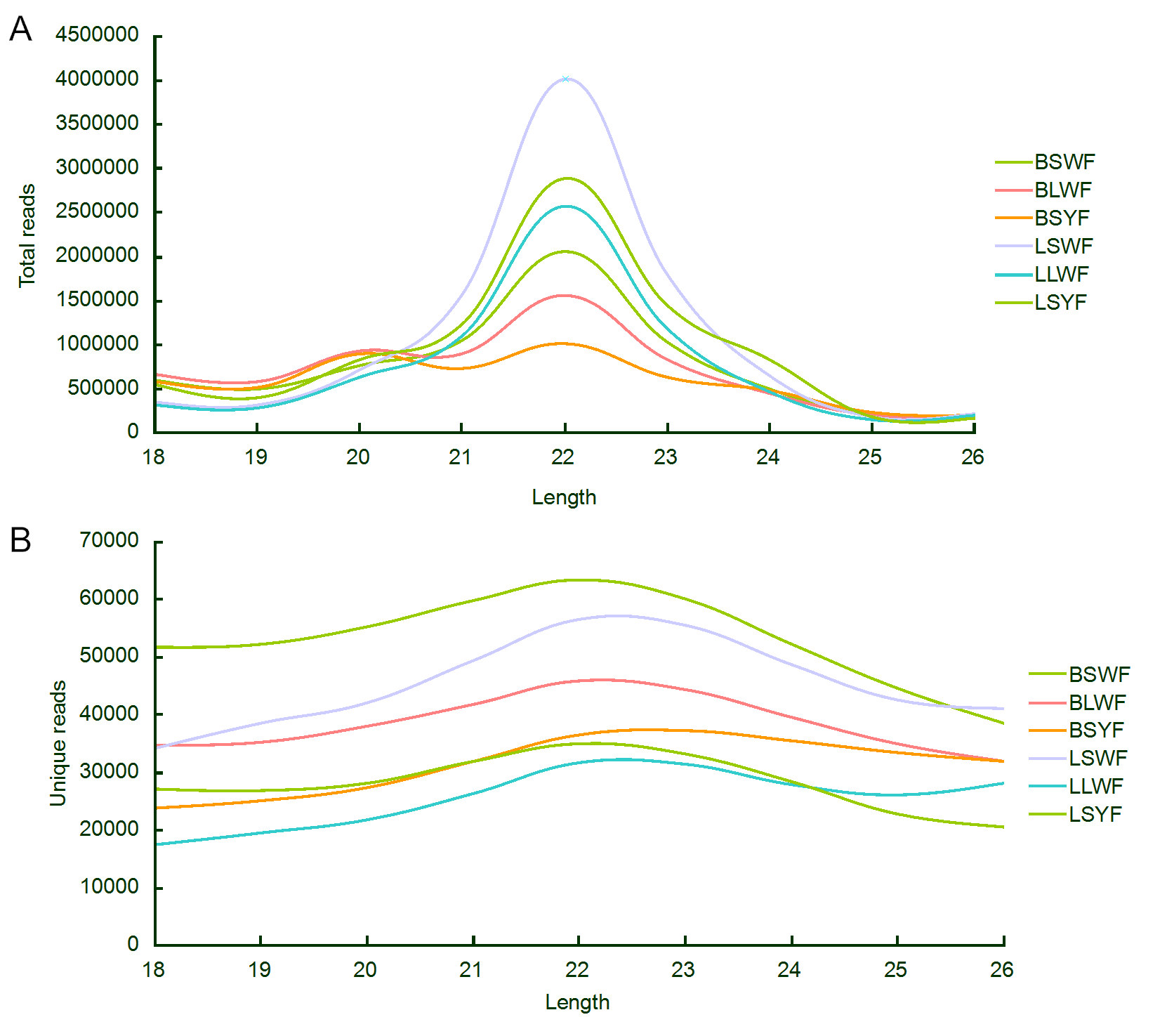

Supplement: Figure S4 [file Figure_S4.tif]
